# It works - but how? Influence of a Recovery-oriented concept on coercive measures on acute psychiatric wards

**DOI:** 10.1192/j.eurpsy.2023.103

**Published:** 2023-07-19

**Authors:** L. Mahler

**Affiliations:** ^1^Psychiatry and Psychotherapy, Clinics in the Theodor-Wenzel-Werk; ^2^Psychiatry and Psychotherapy, Charité University Hospital, Berlin, Germany

## Abstract

**Abstract:**

The United Nations Convention on the Rights of Persons with Disabilities as well as the new guidance on community mental health services recently published by the World Health Organization formulate clear goals for the future of psychiatry and psychosocial support. Innovative concepts of psychiatric care that focus on full participation, recovery-orientation and the prevention of coercion play an important role in achieving these goals. Implementing and scientifically evaluating the effects of such models in mental health services needs to be prioritized in national mental health planning and budgeting decisions.

In 2010, a new recovery-oriented treatment concept, the "Weddinger Modell", was developed and implemented at the Psychiatric University Clinic of the Charité in St. Hedwig Hospital (PUK-SHK). After 13 years of working with the Weddinger Model, there are, in addition to good practical experiences, numerous scientific findings that prove its effectiveness with regard to relationship promotion and reduction of coercive measures on various dimensions. These effects have encouraged many clinics, especially in German-speaking countries, to adopt principles of the "best-practice" model.

The "Weddinger Modell" shows that consistent adjustments of clinic structures and a recovery-oriented attitude of staff lead to a comprehensive improvement of treatment (Mahler et al. 2014; Mahler et al. 2019) and thus to a reduction of coercive measures to an absolute minimum (Czernin et al. 2020; Czernin 2021). Additionally, current studies found that coercive measures can be reduced almost exclusively to the first 24 hours of treatment (Cole et al. 2020). Working with the "Weddinger Modell" shows that coercion can be minimized "without evading responsibility for people with severe mental illness and without denying them responsibility for themselves" (Mahler et al. 2014).

In this lecture, the basic components of the "Weddinger Modell", the attitudes behind it, and the scientific data on effects of the model will be presented and discussed.

**Disclosure of Interest:**

None Declared

